# Electron and Hole Doping Effects on the Magnetic Properties and Band Gap Energy of Ba_2_FeMoO_6_ and Sr_2_FeMoO_6_

**DOI:** 10.3390/molecules30142987

**Published:** 2025-07-16

**Authors:** Angel T. Apostolov, Iliana N. Apostolova, Julia M. Wesselinowa

**Affiliations:** 1 University of Architecture, Civil Engineering and Geodesy, 1046 Sofia, Bulgaria; angelapos@abv.bg; 2 University of Forestry, 1756 Sofia, Bulgaria; inaapos@abv.bg; 3 Faculty of Physics, Sofia University “St. Kliment Ohridski”, J. Bouchier Blvd. 5, 1164 Sofia, Bulgaria

**Keywords:** Ba_2_FeMoO_6_ and Sr_2_FeMoO_6_, ion doping, magnetization, Curie temperature, band gap, s-d model, Green’s function theory

## Abstract

Using the s-d model and Green’s function theory, we investigated for the first time the electron and hole doping effects on the magnetic and optical properties of the double perovskites ${\mathrm{Ba}}_{2}{\mathrm{FeMoO}}_{6}$ (BFMO) and ${\mathrm{Sr}}_{2}{\mathrm{FeMoO}}_{6}$ (SFMO). Our aim was to find the doping ions that lead to an increase in Curie temperature $T_{C}$. On the basis of a competition mechanism between spin exchange and s-d interactions, we explain at a microscopic level the decrease in magnetization *M* and band gap energy $E_{g}$, as well as the increase in $T_{C}$ of BFMO and SFMO through substitution with rare earth ions at the Ba(Sr) sites. The influence of doping with K at the Ba(Sr) and Co at the Fe sites on the magnetic properties and the band gap is also discussed. A very good qualitative coincidence with the existing experimental data was observed. Moreover, we found that both *M* and $T_{C}$ decrease with decreasing the size of BFMO and SFMO nanoparticles.

## 1. Introduction

In recent decades, magnetic double perovskites with the general formula $A_{2}{\mathrm{BB’O}}_{6}$ (where A represents an alkaline-earth or rare-earth (RE) metal, and B, B’ are transition metals) have attracted considerable attention due to their intriguing physical properties [1]. ${\mathrm{Sr}}_{2}{\mathrm{FeMoO}}_{6}$ (SFMO) and ${\mathrm{Ba}}_{2}{\mathrm{FeMoO}}_{6}$ (BFMO) are prominent examples [1,2,3,4,5,6]. Magnetic characterization reveals that these compounds exhibit half-metallic ferrimagnetism, making them highly promising candidates for spintronic applications. Efforts to achieve higher Curie temperatures $T_{C}$ in these materials have motivated the exploration of electron doping as a strategy to strengthen magnetic interactions and thereby enhance $T_{C}$. This issue has been extensively studied by many researchers, both experimentally [7,8,9,10,11,12,13,14,15,16,17] and theoretically [18,19,20,21,22,23,24,25]. However, discrepancies remain regarding the effects of RE ion doping: while some studies report an enhancement of $T_{C}$, others observe a reduction, a point we aim to address. In BFMO, which exhibits a $T_{C}$ of approximately 310 K, the Fe and Mo ions are antiferromagnetically coupled, implying that their spins are aligned in opposite directions. The strength of this coupling is influenced by the Fe-Mo interatomic distance, which is determined by the lattice parameters. A reduction in the lattice parameters leads to a shorter Fe-Mo distance, potentially strengthening the antiferromagnetic coupling. This effect can contribute to an increase in the magnetic ordering temperature [8]. It has been reported that electron doping in SFMO double perovskites, which possess a $T_{C}$ around 400 K, achieved through the substitution of divalent ${\mathrm{Sr}}^{+2}$ ions with trivalent ${\mathrm{La}}^{+3}$ ions, results in a $T_{C}$ enhancement of 70 K at 50% La doping, accompanied by a reduction in magnetization *M* [13]. In contrast, Ghorbani et al. [8] observed a decrease in $T_{C}$ and an increase in *M* upon ${\mathrm{Sm}}^{3+}$ doping in BFMO. For ${\mathrm{Nd}}^{3+}$-doped SFMO, Habib et al. [9] found that the magnetic ordering temperature remained nearly unchanged. Different behavior was reported for Sm-doped SFMO, where $T_{C}$ initially decreased with increasing doping concentration and then rose beyond *x* = 0.15, suggesting that steric effects became more pronounced with decreasing RE ion radius [12]. Sanchez et al. [17] investigated the doped SFMO with both RE ion ${\mathrm{Sm}}^{3+}$ and transition-metal ion ${\mathrm{Ag}}^{+}$ at the ${\mathrm{Ba}}^{2+}$ site, in order to compare the impacts of electron and hole doping on the magnetic properties. The influence of K doping on $T_{C}$ in BFMO was studied by Kim et al. [11], who reported a reduction in *M* and $T_{C}$. Similarly, V doping at the Mo-site in BFMO led to a decrease in $T_{C}$ and *M* [26].

The ability to modify the band gap in SFMO and BFMO through a wide variety of dopant elements and concentrations renders these materials promising candidates for solar cell applications [27,28,29].

Let us emphasize that the properties of SFMO and BFMO nanostructures, which have been studied in recent years [30,31,32,33,34], could have been changed and therefore different from those of bulk materials. The magnetic properties of SFMO nanoscale powders where superparamagnetism is observed were studied by Kalanda et al. [30] and Suominen et al. [31]. The magnetization of magnetically inhomogeneous SFMO NPs was described by Suchaneck et al. [32]. The size dependence of electronic and magnetic properties of SFMO was investigated by Li et al. [33]. Naushahi et al. [34] reported increased $T_{C}$ and *M* by modifying the Fe/Mo ratio in SFMO thin films.

In this work, we investigate, for the first time, the effects of ion doping on the magnetic properties and band gap energy of BFMO and CFMO, employing a microscopic model and Green’s function theory, which accounts for the interplay between spin exchange and s-d interactions. The properties of BFMO and SFMO nanoparticles are also discussed.

## 2. Model and Method

The Mo electrons are delocalized and facilitate ferromagnetic coupling between ${\mathrm{Fe}}^{3+}$ ions through hybridization with the unoccupied spin-down Fe ${\mathrm{3dt}}_{2g}$ orbitals. The magnetic behavior of Mo-based double perovskites can be described within the framework of the s-d exchange model:

(1) $$H=H_{sp}+H_{el}+H_{sp-el}.$$

$H_{sp}$ is the modified Heisenberg lHamiltonian:

(2) $$\begin{array}{ccc} H_{sp} & = & -\sum_{ij}J_{ij}^{Fe-Fe}S_{i}^{Fe}\cdot S_{j}^{Fe}-\sum_{ij}\left(1-x\right)J_{ij}^{Fe-Mo}S_{i}^{Fe}\cdot S_{j}^{Mo}\\ & - & \sum_{ij}xJ_{ij,d}^{Fe-Mo}S_{i}^{Fe}\cdot S_{j}^{Mo}-D\sum_{i}{\left(S_{i}^{zFe}\right)}^{2}-g\mu_{B}h\sum_{i}S_{i}^{zFe}. \end{array}$$

**Figure 1:**
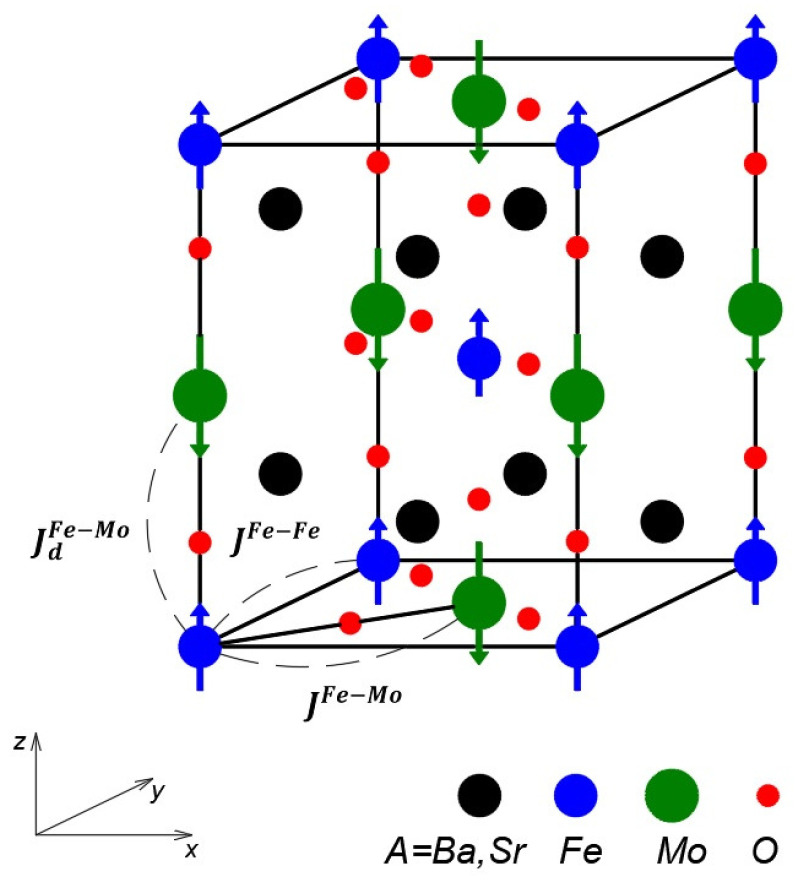
The crystal structure of Mo-based double perovskites BFMO (SFMO) and the exchange interactions.

The first two terms account for the exchange interactions within the Fe and Fe-Mo ions into the xy-plane, respectively, while the third term describes the inter-sublattice coupling between the Fe and Mo layers (see Figure 1). Exchange interaction strengths are given by $J_{ij}$, and the single-ion anisotropy parameters by $D_{i}$. The index *d* denotes $J_{ij}$ by ion doping. The system is subjected to an external magnetic field *h*, and *x* corresponds to the doping concentration. In SFMO and BFMO compounds, the ${\mathrm{Fe}}^{3+}$ and ${\mathrm{Mo}}^{5+}$ ions possess spin values of *S* = 5/2 (${\mathrm{3d}}^{5}$) and *S* = 1/2 (${\mathrm{4d}}^{1}$), respectively, resulting in net magnetic moments of 4.0 and −0.5$\mu_{B}$. The Fe and Mo ions are coupled antiferromagnetically via oxygen through a superexchange mechanism.

Depending on the strain introduced by the dopant ions, the exchange coupling $J_{d}$ may become either greater or smaller than the unperturbed exchange parameter *J*. This treatment is justified, as the exchange interaction $J_{ij}\equiv J\left(r_{i}-r_{j}\right)$ is inversely related to the distance between interacting spins and the lattice constants. As a result, changes in lattice symmetry and the coordination number of next-nearest neighbors significantly affect the interaction strength. This principle extends similarly to all other exchange interaction parameters.

The Hamiltonian $H_{el}$ represents the conduction band electrons and is expressed as

(3) $$H_{el}=\sum_{ij\sigma }t_{ij}c_{i\sigma }^{+}c_{j\sigma },$$

where $t_{ij}$ is the hopping integral characterizing the probability amplitude for an electron to move from site *i* to site *j*. The Fermi operators $c_{i\sigma }^{+}$ and $c_{i\sigma }$ correspond to the creation and annihilation of an electron with spin σ at the *i*-th and *j*-th lattice sites, respectively.

The Hamiltonian $H_{sp-el}$ describes the s-d interaction between localized magnetic moments and itinerant conduction electrons. This interaction plays a crucial role in mediating magnetic ordering in systems such as doped double perovskites, where both localized and delocalized electrons coexist. The expression is given by

(4) $$H_{sp-el}=-\sum_{i}\left(1-x\right)I_{i}S_{i}s_{i},$$

Here, $I_{i}$ denotes the intra-atomic exchange coupling constant, $S_{i}$ is the spin operator associated with the localized moment (typically on Fe or a magnetic ion), and $s_{i}$ is the spin operator corresponding to the conduction electron at site *i*. The prefactor (1 − *x*) reflects the effect of doping, where *x* represents the concentration of nonmagnetic or substituting ions, reducing the number of magnetic interactions. The conduction electron spin operator $s_{i}$ can be expressed in terms of fermionic creation and annihilation operators. For example, the spin raising operator is: $s_{i}^{+}=c_{i+}^{+}c_{i-}$, and the *z*-component is: $s_{i}^{z}=\left(c_{i+}^{+}c_{i+}-c_{i-}^{+}c_{i-}\right)/2$, where $c_{i\sigma }^{+}$ and $c_{i\sigma }$ are the creation and annihilation operators for an electron with spin σ at the *i*-th site. This exchange term effectively aligns or anti-aligns the spins of conduction electrons with the localized spins depending on the sign and magnitude of $I_{i}$, influencing the system’s magnetic ground state and transport properties.

In SFMO and BFMO materials, the electronic band gap—an important parameter governing their semiconducting and magnetic behavior—is observed to be in the range from 2 to 2.1 eV [35]. This gap arises from the electronic structure in which the minority-spin (low-spin) ${\mathrm{Mo}}^{5+}$ ions, with a ${\mathrm{4d}}^{1}$ configuration, contribute states to the conduction band, while the majority-spin (high-spin) ${\mathrm{Fe}}^{3+}$ ions (${\mathrm{3d}}^{5}$) form the valence band. To quantify the band gap energy $E_{g}$ in numerical calculations, the following expression is used:

(5) $$E_{g}=\epsilon^{+}\left(k=0\right)-\epsilon^{-}\left(k=k_{\sigma }\right),$$

where $\epsilon^{+}\left(k=0\right)$ represents the energy at the top of the valence band (typically at the center of the Brillouin zone), and $\epsilon^{-}\left(k=k_{\sigma }\right)$ corresponds to the energy at the bottom of the conduction band, possibly occurring at a different point $k_{\sigma }$ in reciprocal space. The notation accounts for the possibility of an indirect band gap, where the momentum values associated with the conduction band minimum and valence band maximum differ.

The electron energy levels in these systems are described by the expression:

(6) $$\epsilon_{ij}^{\pm }=\epsilon_{ij}-\frac{\sigma }{2}\left(1-x\right)IM$$

where $\epsilon_{ij}$ represents the band electron energies in the paramagnetic state, and σ = ±1 corresponds to the spin orientation. *M* is the magnetization of the sample, and *x* denotes the doping concentration. This formula captures how the exchange interaction shifts the energy levels depending on the spin polarization and doping, effectively splitting the bands in the ferromagnetic phase. These energies are obtained from the poles of the single-particle Green’s function, $g_{ij\sigma }=\ll c_{i\sigma };c_{j\sigma }^{+}\gg$, which describes the propagation of electrons with spin σ in the lattice and includes the effects of electron correlations and spin interactions.

To fully characterize the magnetic state of double perovskites, the magnetization *M* in the ferromagnetic regime is calculated through a self-consistent expression, reflecting the collective alignment of localized spins and conduction electrons that underlies the system’s magnetic ordering:

(7) $$\begin{array}{ccc} M & = & \langle S^{z}\rangle =\frac{1}{N^{2}}\sum_{i,j}[\left(S+0.5\right)\coth\left[\left(S+0.5\right)\beta E_{ij}\right]\\ & - & 0.5\coth(0.5\beta E_{ij})], \end{array}$$

where 〈...〉 is the thermodynamic mean value.

The spin-wave energy $E_{ij}$ is calculated from the Green’s function

(8) $$G_{ij}\left(t\right)=\ll S_{i}^{+}\left(t\right);S_{j}^{-}\gg$$

using the method of Tserkovnikov [36] taking into account the transverse correlation functions $\langle S_{i}^{-}S_{j}^{+}\rangle$ and decoupling the longitudinal ones $\langle S_{i}^{z}S_{j}^{z}\rangle \to \langle S_{i}^{z}\rangle \langle S_{j}^{z}\rangle$.

## 3. Numerical Results and Discussion

The structure of BFMO or SFMO is cubic Fm$\bar{3}$m above $T_{C}$ and tetragonal I4/mmm below $T_{C}$. In the ordered double perovskite BFMO or SFMO, the transition metal sublattice sites are occupied alternately by Fe and Mo ions with an antiferromagnetic coupling between them (see Figure 1).

The calculations of magnetic properties of BFMO or SFMO are performed based on the JAVA software platform and using a self-consistent iterative procedure. A self-consistent iterations method was used. The starting parameters for the iterations are the model parameters given below at *T* = 0 K. In any subsequent calculation of the input data, the results of the previous calculation were used. The calculations continue until the difference between two consecutive iterations does not exceed a predetermined small enough value. The numerical calculations are made using the following model parameters: $J^{Fe-Fe}$ = 7.69 meV [37], $J^{Fe-Mo}$ = −18 meV [25], $D^{Fe}$ = −0.0013 meV [37], *I* = 0.2 eV [25], $T_{C}\left(SFMO\right)$ = 400 K, $T_{C}\left(BFMO\right)$ = 310 K.

### 3.1. Properties of K-Doped BFMO and SFMO at the Ba(Sr) Site

**Figure 2:**
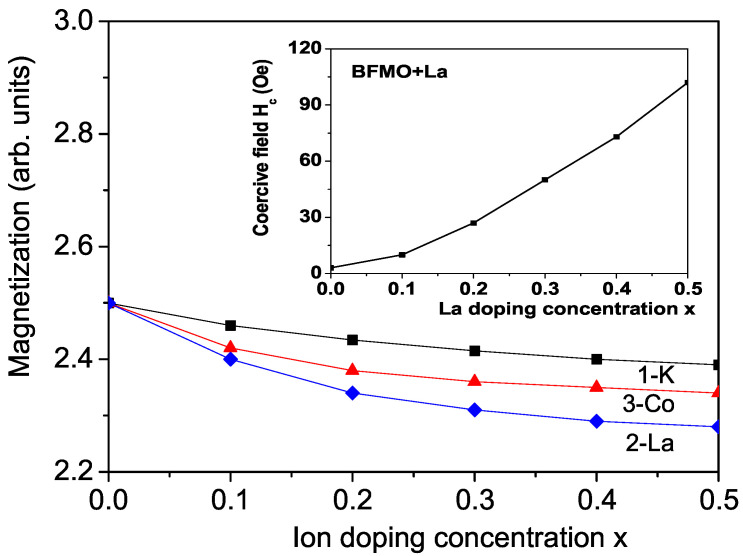
Ion doping concentration dependence of the magnetization in BFMO for *T* = 10 K and for different ions at the ${\mathrm{Ba}}^{2+}$ site: (1) $K^{+}$ ($J_{d}^{Fe-Fe}=0.9J^{Fe-Fe}$, $J_{d}^{Fe-Mo}=0.9J^{Fe-Mo}$, $I_{d}=0.9I$); (2) ${\mathrm{La}}^{3+}$ ($J_{d}^{Fe-Fe}=0.7J^{Fe-Fe}$, $J_{d}^{Fe-Mo}=0.7J^{Fe-Mo}$, $I_{d}=0.7I$), and at the ${\mathrm{Fe}}^{3+}$ site (3) ${\mathrm{Co}}^{2+}$ ($J_{d}^{Fe-Fe}=0.8J^{Fe-Fe}$, $J_{d}^{Fe-Mo}=0.8J^{Fe-Mo}$, $I_{d}=1.1I$). The values are valid for all Figures. Inset: La doping concentration dependence of the coercive field $H_{c}$ in La-doped BFMO.

**Figure 3:**
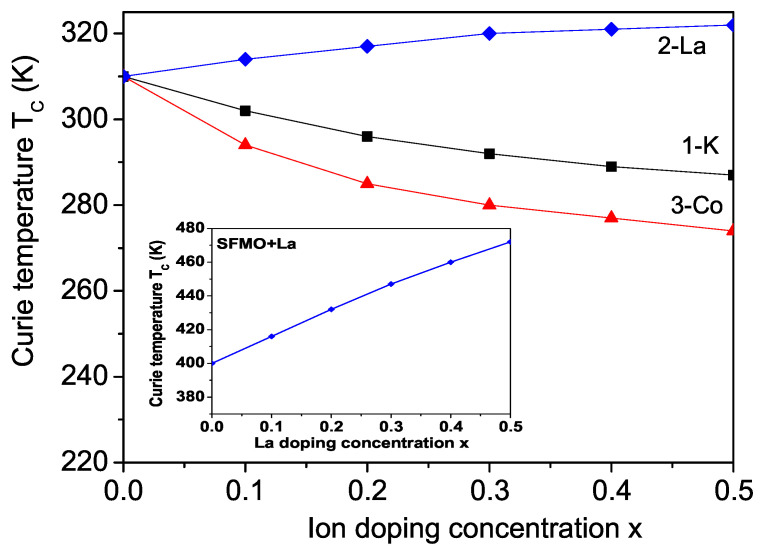
Ion doping concentration dependence of the Curie temperature $T_{C}$ in BFMO for different ions at the ${\mathrm{Ba}}^{2+}$ site: (1) $K^{+}$; (2) ${\mathrm{La}}^{3+}$, and at the ${\mathrm{Fe}}^{3+}$ site (3) ${\mathrm{Co}}^{2+}$. Inset: La doping concentration dependence of the Curie temperature $T_{C}$ in SFMO.

Firstly we will consider the doping dependence of the spontaneous magnetization *M* and Curie temperature $T_{C}$ in $K^{+}$-doped (hole-doped) BFMO and SFMO at the ${\mathrm{Ba}}^{2+}$ or ${\mathrm{Sr}}^{2+}$ site, i.e., there is a hole injection, which may help balance the charge distribution in the material. The doped holes enter selectively the spin-down band. $K^{+}$ doping leads to the creation of holes at B and B’ sites in BFMO or SFMO which can change the charge difference between the cations at these two sites [11,38]. The substitution of $K^{+}$ for ${\mathrm{Ba}}^{2+}$(${\mathrm{Sr}}^{2+}$) ions may induce the decrease of the chemical valence at A-site, which may be compensated by the increase of the chemical valence at B/B’-sites, i.e., the decrease of the chemical valence at ${\mathrm{Ba}}^{2+}$(${\mathrm{Sr}}^{2+}$)/$K^{+}$ site results in the variation of the chemical valence of Fe or Mo. X-ray photoelectron spectroscopy results in K doped BFMO or SFMO have displayed mixed valence at the B and B’ sites, namely ${\mathrm{Fe}}^{3+}$/${\mathrm{Fe}}^{4+}$ and ${\mathrm{Mo}}^{5+}$/${\mathrm{Mo}}^{4+}$, confirmed by the variation of the averaged bond-length of Fe-O and Mo-O. The ionic radius of $K^{+}$ is 1.52 $\dot{A}$, whereas of ${\mathrm{Ba}}^{2+}$ it is 1.49 $\dot{A}$, and of ${\mathrm{Sr}}^{2+}$ is 1.44 $\dot{A}$, i.e., the lattice parameters increase with the increase in K ion doping, i.e., there appears a tensile strain. In our model this means that by K ion doping, we have to use the relations between the spin exchange interaction and s-d interaction constants in the doped and undoped states $J_{d}<J$, $I_{d}<I$. We observe a decrease in the spontaneous magnetization *M* and Curie temperature $T_{C}$ in BFMO (see Figure 2 and Figure 3, curve 1), as reported experimentally by Kim et al. [11] in K-doped BFMO. The decrease in the magnetization *M* may be attributed to the decrease in $J_{d}$ (due to the tensile strain) as well to the increase in the holes in the material and blocking transfer electrons between grains due to the holes, i.e., a decrease in $I_{d}$. The decrease in the Curie temperature $T_{C}$ with K doping is associated with carrier doping. We observe the same behavior, a decrease in M(x) and $T_{C}\left(x\right)$ in K-doped SFMO. A reduction in Curie temperature and magnetization is reported also in Na-doped BFMO at the Ba site [39].

**Figure 4:**
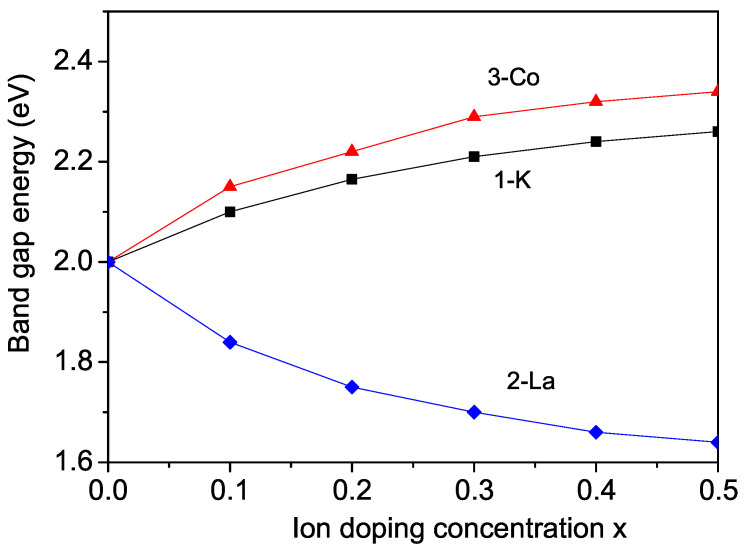
Ion doping concentration dependence of the band gap energy $E_{g}$ in BFMO for different ions at the ${\mathrm{Ba}}^{2+}$ site: (1) $K^{+}$; (2) ${\mathrm{La}}^{3+}$, and at the ${\mathrm{Fe}}^{3+}$ site (3) ${\mathrm{Co}}^{2+}$.

According to the literature, the small band gap in SFMO or BFMO is a critical factor in photocatalytic activity. Using Equations (5) and (6) as well Equation (7) self-consistently we have calculated the band gap energy $E_{g}$. The K doping concentration dependence of $E_{g}$ in BFMO is shown in Figure 4, curve 1. It can be seen that $E_{g}$ increases with the increase in K doping. Unfortunately, there are not experimental data for $E_{g}\left(x\right)$ for K-doped BFMO.

### 3.2. Properties of La-Doped BFMO and SFMO at the Ba(Sr) Site

Next, we will study the effects of ${\mathrm{La}}^{3+}$ ion doping (electron doping in the conducting band) at the ${\mathrm{Sr}}^{2+}$ site in SFMO or at the ${\mathrm{Ba}}^{2+}$ site in BFMO. Let us note that ${\mathrm{La}}^{3+}$ ion has an empty 4f orbital (${\mathrm{4f}}^{0}$), where the electrons are paired, and thus it has diamagnetic properties with spin value *S* = 0. From band calculations is observed that the doped electrons occupy mainly the down-spin Mo 4d band [40]. The ionic radius of ${\mathrm{La}}^{3+}$ ($1.36\;\dot{A}$) is smaller than that of ${\mathrm{Sr}}^{2+}$ ($1.44\;\dot{A}$) and ${\mathrm{Ba}}^{2+}$ ($1.49\;\dot{A}$), i.e., the lattice parameters should be reduced and a compressive strain should occur. However, experimentally an increase in the cell volume is observed for the lattice parameters in La-doped BFMO [15]. The difficulty arises because in these doped materials, the La substitution may not only provide carriers to the conduction band but also promote a structural distortion owing to the different ionic radii of Sr(Ba)^2+^ and ${\mathrm{La}}^{3+}$ ions. For the La ion doping, a strong anti-site disorder between the Fe and Mo ions also appears, $Fe_{Mo}-Mo_{Fe}$ [41]. It arises due to exchange of Fe and Mo moments among the B and B’ sites of the double perovskite structure [8]. This assumption is experimentally confirmed in La-doped SFMO by Muthuselvam et al. [42] using magnetic and transport measurements and by Sanchez et al. [43] by neutron magnetic scattering and neutron powder diffraction techniques. The observed decrease in magnetization *M* and increase in critical temperature $T_{C}$ in these materials is explained in terms of the decreasing site ordering of ${\mathrm{Fe}}^{3+}$ and ${\mathrm{Mo}}^{5+}$ ions (i.e., increase in anti-site defects). Let us note that anti-site disorder is not observed by Chmaissem et al. [5] using neutron powder diffraction in BFMO, whereas it is reported by many authors in pure and ion-doped BFMO and SFMO, for example Chang et al. [44], Zhang et al. [16], Ghorbani et al. [8], Navarro et al. [13,14], Yang et al. [41]. Moreover, upon the ${\mathrm{La}}^{3+}$ for ${\mathrm{Ba}}^{2+}$ substitution, extra electrons enter the Mo-dominated conduction band, which means that Mo is partially reduced from ${\mathrm{Mo}}^{5+}$ ($0.75\;\dot{A}$) to ${\mathrm{Mo}}^{4+}$ ($0.79\;\dot{A}$), and as the latter ion has a greater radius an increase in the unit cell volume occurs. In our model this means that the exchange interaction constants at the doped states, denoted as $J_{d}$ are smaller compared to those in the undoped ones *J*, i.e., $J_{d}<J$. The most experimental data show that whereas the magnetization *M* decreases the Curie temperature $T_{C}$ increases enhancing the La doping concentration *x*. This could be explained considering that there is a competing mechanism between the exchange interaction *J* and the s-d interaction *I*. Let us note that Frontera et al. [45] reported an enhancement of the $\langle d_{(Fe,Mo)-O}\rangle$ bond distance in the La series, which signals an effective electron doping in the Fe-Mo sublattice and thus the filling up of the conducting band and a simultaneously rising of the density of states at the Fermi level. The La ions lead to the electron filling of the conduction band. Therefore, we have to take additively to $J_{d}<J$ the following relation $I_{d}>I$. All equations must be calculated self-consistently. As a consequence, we obtain a decrease in the magnetization *M* with an increase in the ${\mathrm{La}}^{3+}$ electron doping concentration *x*. Moritomo et al. [46] and Wojcik et al. [47] reported an enhanced magnetic moment at the Mo site in SFMO. Moreover, the doped electrons are considered to occupy mainly the down-spin Mo 4d band, which also leads to a decrease in magnetization *M* (see also Equation (7)). The result of M(x) for La-doped BFMO is presented in Figure 2, curve 2. The same result is observed for SFMO. A decrease in M(x) is reported experimentally in La-doped BFMO [15] and SFMO [13,14,17,46,48,49], as well as in ${\mathrm{Sm}}^{3+}$-, ${\mathrm{Nd}}^{3+}$- or ${\mathrm{Pr}}^{3+}$-doped SFMO and BFMO [8,10,12,40,50,51,52]. Let us emphasize that doping with these RE ${\mathrm{ions—Sm}}^{3+}$, ${\mathrm{Nd}}^{3+}$ or ${\mathrm{Pr}}^{3+}$ (contrary to the La ion with ${\mathrm{4f}}^{0}$ and zero magnetic moment)—introduces f-electrons, which are localized and contribute to the total moment. These RE ions are paramagnetic due to unpaired electrons in the 4f subshells. However, although these elements contribute to the total magnetic moment, this does not change the discussion made above regarding ${\mathrm{La}}^{3+}$. The RE doping reduces the lattice parameter and bond lengths of RE/Ba-O and Fe/Mo-O, but consequently, it enhances the ferrimagnetic transition temperature $T_{C}$. On the other hand, it increases gradually the probability of Fe-Mo anti-site disorder which is the main reason for the decrease in the magnetization.

Next, we will consider the Curie temperature $T_{C}$ in La-doped SFMO and BFMO. In order to enhance $T_{C}$ of SFMO or BFMO, as reported by extensive experimental data, the injection of electrons into the conduction band by appropriate doping should be carried out. This can be achieved by the substitution of trivalent ${\mathrm{La}}^{3+}$ for the divalent ${\mathrm{Sr}}^{2+}$ or ${\mathrm{Ba}}^{2+}$ in SFMO or BFMO. We calculated the dependence of $T_{C}$ on the doping concentration *x*, $T_{C}\left(x\right)$ in BFMO, and observed a substantial increase in $T_{C}$ due to the correlation effects on Fe and Mo sites, caused by the effect of the conduction band-filling when doping with La and enhancing the s-d interaction $I_{d}$ in the doped materials. We take into account in our calculations the interaction between localized Fe-spins and conduction Mo-electrons via a double-exchange-type mechanism. We also include the electronic correlations among the conduction electrons within a mean-field approximation. Our results show that the Curie temperature increases when La concentration increases in the La-doped BFMO compound (see Figure 3, curve 2). For *x* = 0.5, we observe $T_{C}$ = 322 K, i.e., an increase in 12 K, which is in very good quantitative agreement with the reported result of Yang et al. [15]. They obtained an increase in Curie temperature $T_{C}$ in La-doped BFMO from 316 K for *x* = 0 to 326 K for *x* = 0.5. We also calculated the dependence of $T_{C}$ in La-doped SFMO. The result is shown in the inset in Figure 3. For *x* = 0.5, we observe a Curie temperature of $T_{C}$ = 472 K, i.e., an increase of 72 K, which is in very good quantitative agreement with the reported result of Navarro et al. [13,14]. They observed an increase of 70 K for *x* = 0.5, which is much more compared to the enhanced $T_{C}$ in La-doped BFMO. The good quantitative agreement is evidence for the accuracy of the proposed model and the approximations made by the calculations. The observed increase in $T_{C}$ through the doping of SFMO and BFMO with rare earth ions, i.e., by electron doping, agrees with the experimental data of Aguilar et al. [49], Guzman et al. [53], Frontera et al. [45], Kahoul et al. [48] in La-doped SFMO. An increase in $T_{C}$ is observed in Nd-doped SFMO [40,51,52] and Pr-doped BFMO [10]. Zhang et al. [12] reported an increased $T_{C}$ in Sm-, Nd-, Ce-, and Pr-doped SFMO and a reduced $T_{C}$ value for the Eu-doped compound. Hoffmann et al. [54] have shown that oxygen vacancies in SFMO can increase $T_{C}$, although *M* is reduced at the same time. Let us note that Sanchez et al. [17] and Ghorbani et al. [8,50] found a decrease in $T_{C}$ in La- and Sm-doped SFMO and BFMO, respectively, in disagreement with our results.

In summary, the increase in Curie temperature $T_{C}$ with the increase in rare earth ion doping concentrations in SFMO and BFMO is attributed to the effect of the conduction band-filling; it originates from electron doping effects in addition to ionic size effects. The reduction in the spontaneous magnetization *M* and the increase in the Curie temperature $T_{C}$ can be explained with the competition mechanism between the spin exchange interaction *J* and the s-d interaction *I* in the doped compounds.

The coercive field $H_{c}$ in La-doped BFMO at the Ba site is also calculated. $H_{c}$ increases with the increase in La dopant. The result is shown in the inset in Figure 2. A similar behavior of the La doping dependence of the coercive field $H_{c}\left(x\right)$ is reported by Aguilar et al. [49] in La-doped SFMO.

From Equations (5)–(7) we evaluated self-consistently the band gap energy $E_{g}$ as a function of the La ion doping concentration *x* in BFMO. The result is shown in Figure 4 (curve 2). $E_{g}$ decreases with the increase in La dopant *x*, which is due to the competition mechanism between the exchange interactions and s-d interactions, due to the increase in the s-d interaction in the doped states $I_{d}$. We observe a similar behaviour of $E_{g}\left(x\right)$ by doping with other rare earth ions. The decrease in the band gap energy $E_{g}$ is in agreement with the experimental data for rare earth ions doped with BFMO and SFMO [55,56].

### 3.3. Properties of Co-Doped BFMO and SFMO at the Fe Site

Let us note that the substitution of ions can also be made at the Fe site in the Mo-based double perovskites, BFMO or SFMO, both with divalent ions (such as Co and Mn) or trivalent cations (such as Al, Cr, and Ga) [44,57,58,59,60,61,62,63]. Therefore, we studied Co doping in BFMO. Both the experiments and calculations of Chang et al. [44] reveal that the Co ion takes a +2 valence (${\mathrm{3d}}^{7}$ configuration) in Co-doped SFMO. The shrinking Mo-O bond length indicates an increasing average valence of the Mo ion, i.e., from +5(${\mathrm{3d}}^{1}$) to +6(${\mathrm{3d}}^{0}$) with the increase in Co dopant. This is necessary to achieve electroneutrality. The ionic radius of the doped ${\mathrm{Co}}^{2+}$ ion ($0.79\;\dot{A}$) is greater than that of the host ${\mathrm{Fe}}^{3+}$ ion ($0.645\;\dot{A}$), i.e., when a tensile strain appears, the lattice parameters and the cell volume are enhanced. This agrees with the experimental data for Co-doped SFMO of Chang et al. [44]. The authors show that the cell volume continuously increases as ${\mathrm{Co}}^{2+}$ content increases from 0 to 0.9, from *V* = 0.245658 to $0.246638\;{\dot{A}}^{3}$, and the lattice parameter *c* from *c* = 0.79067 to $0.79474\;\dot{A}$. This increase leads to the following relations between the doped and undoped exchange interaction and s-d interaction parameters: $J_{d}<J$ and $I_{d}<I$. Let us note that the situation here is different compared to that of La ion doping at the Ba(Sr) site as discussed in Section 3.2. There is a substitution of a magnetic ion with another one. Co doping does not create a competing mechanism. The magnetic order changes from ferrimagnetic to antiferromagnetic [58]. Magnetization *M* and the Curie temperature $T_{C}$ decrease with the increase in Co doping concentration (see Figure 2 and Figure 3, curve 3, respectively). A similar behaviour of M(x) and $T_{C}\left(x\right)$ in Co-doped SFMO was reported experimentally by Chang et al. [44]. A drop of *M* and $T_{C}$ was also obtained in Mn-, Ga-, Al-, In- and V-doped SFMO at the Fe site in [60,61,62,63,64,65]. Sriti et al. [66] observed a decrease in ferromagnetism and Curie temperature through the substitution of various divalent (Mg, Zn, Ca) and trivalent (Cr, Mn, In, Y) cations for Fe in BFMO. Unfortunately, there are no experimental data for the magnetic behavior of Co-doped BFMO. But Mahmood [67] reported for the double perovskite ${\mathrm{Ba}}_{2}{\mathrm{CoMoO}}_{6}$ a decrease of 12 K for $T_{C}$ in comparison to BFMO. Therefore, we assume that our results are correct.

The Co doping concentration dependence of the band gap energy $E_{g}$ in BFMO is calculated from Equations (5)–(7). It must be noted that when increasing the substitution of Co for Fe, the behaviour changes from a typical half-metal to a semiconductor behavior. The result for $E_{g}\left(x\right)$ is depicted in Figure 4 (curve 3). It can be seen that the band gap energy $E_{g}$ increases with the increase in Co doping concentration *x*, in agreement with the experimental results of Wang et al. [68]. Large band gaps of these materials are possible for their application in photocatalysis.

### 3.4. Properties of BFMO and SFMO Nanoparticles

Finally, we will study the magnetization *M* and Curie temperature $T_{C}$ of BFMO nanoparticles (NPs) taking into account surface and size effects. The NP is defined by fixing the origin at an arbitrary spin at the center of the particle. The remaining spins are arranged within the particle on shells (cuboctahedra surrounded by triangular and square walls). The shells are labeled with *n* = 0, 1, …, *N*, where *n* = 0 represents the central spin and *n* = *N* corresponds to the surface shell of the system. Due to defects, uncompensated spins and surface disorder, the exchange interaction constant at the surface, denoted as $J_{s}$, differs from that in the bulk, *J*. This difference can result in $J_{s}$ being either smaller or larger than *J*. For $J_{s}>J$ the magnetization increases with the decrease in NP size. We chose the case $J_{s}<J$ because the experimental data for BFMO NPs show that the magnetization decreases with th decrease in NP size. The s-d interaction constant on the surface $I_{s}$ can be also different compared to the bulk one *I*.

**Figure 5:**
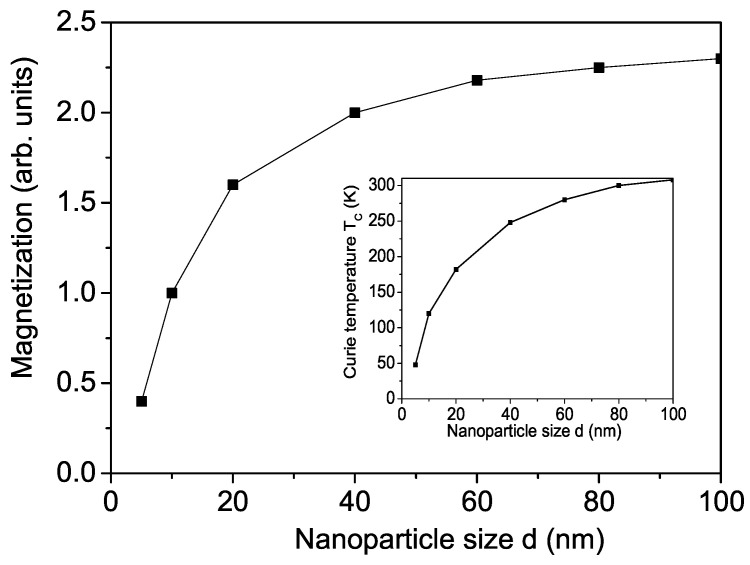
Size dependence of the magnetization *M* in BFMO. Inset: Size dependence of the Curie temperature $T_{C}$ in BFMO.

The size effect of the magnetization *M* for BFMO is shown in Figure 5 with the model parameters $J_{s}=0.8J$, $I_{s}=0.8I$. The spontaneous magnetization *M* decreases as the NP size *d* decreases. The Curie temperature $T_{C}$ is reduced too (see inset in Figure 5). Below *d* ≈ 5 nm we obtain a superparamagnetism and the magnetization vanishes. Unfortunately, there are no experimental data for M(d) and $T_{C}\left(d\right)$ in BFMO. We observed a similar reduction in *M* and $T_{C}$ in SFMO, in agreement with the experimental results in [31,32]. Let us note that the values of the magnetization and the Curie temperature are very sensitive on the surface interaction constants.

It can be concluded that, due to the smaller $T_{C}$ values observed in BFMO and SFMO NPs, doping with various ions is very important in order to enhance the Curie temperature. A high Curie temperature makes them promising candidates for magnetocaloric applications and for spintronics. The magnetic and optical properties of electron- and hole-doped SFMO and BFMO NPs will be investigated in a future paper.

## 4. Conclusions

In conclusion, we investigated theoretically the electron and hole doping effects on the magnetization *M*, the Curie temperature $T_{C}$, and the band gap $E_{g}$ of BFMO and SFMO. On the basis of a competing mechanism between the spin exchange and s-d interactions, we explained, at a microscopic level, the decrease in *M* and $E_{g}$, as well as the increase in $T_{C}$ by substitution with rare earth ions (La, Sm, Nd) at the Ba(Sr) sites. The observation that $T_{C}$ can be substantially enhanced in BFMO and SFMO makes them promising candidates for magnetocaloric applications and may be of relevance for technological applications of these materials in advanced spin devices.

The influence of doping with K at the Ba(Sr) sites or Co at the Fe sites on the magnetic properties—spontaneous magnetization *M* and Curie temperature $T_{C}$—as well as on the band gap energy $E_{g}$ was also discussed. It was found that *M* and $T_{C}$ decrease, whereas $E_{g}$ increases by doping with K and Co ions. It can be concluded that more experimental and theoretical studies are needed to clarify the discrepancies in the reported properties of ion-doped Mo-based double perovskites.

We also investigated the magnetic properties of BFMO and SFMO NPs and found that both the magnetization and Curie temperature decrease with the decrease in NP size. 

## Data Availability

Derived data supporting the findings of this study are available from the corresponding author upon reasonable request.
